# ESR1 inhibits hCG-induced steroidogenesis and proliferation of progenitor Leydig cells in mice

**DOI:** 10.1038/srep43459

**Published:** 2017-03-07

**Authors:** Yeong Seok Oh, Il Kyoo Koh, Bomi Choi, Myung Chan Gye

**Affiliations:** 1 Department of Life Science and Research Institute for Natural Sciences, Hanyang University, Seoul 04763, Korea

## Abstract

Oestrogen is an important regulator in reproduction. To understand the role of oestrogen receptor 1 (ESR1) in Leydig cells, we investigated the expression of ESR1 in mouse Leydig cells during postnatal development and the effects of oestrogen on steroidogenesis and proliferation of progenitor Leydig cells (PLCs). In Leydig cells, the ESR1 expression was low at birth, increased until postnatal day 14 at which PLCs were predominant, and then decreased until adulthood. In foetal Leydig cells, ESR1 immunoreactivity increased from birth to postnatal day 14. These suggest that ESR1 is a potential biomarker of Leydig cell development. In PLCs, 17β-estradiol and the ESR1-selective agonist propylpyrazoletriol suppressed human chorionic gonadotropin (hCG)-induced progesterone production and steroidogenic gene expression. The ESR2-selective agonist diarylpropionitrile did not affect steroidogenesis. In PLCs from *Esr1* knockout mice, hCG-stimulated steroidogenesis was not suppressed by 17β-estradiol, suggesting that oestrogen inhibits PLC steroidogenesis via ESR1. 17β-estradiol, propylpyrazoletriol, and diarylpropionitrile decreased bromodeoxyuridine uptake in PLCs in the neonatal mice. In cultured PLCs, 17β-estradiol, propylpyrazoletriol, and diarylpropionitrile reduced hCG-stimulated *Ki67* and *Pcna* mRNA expression and the number of KI67-positive PLCs, suggesting that oestrogen inhibits PLC proliferation via both ESR1 and ESR2. In PLCs, ESR1 mediates the oestrogen-induced negative regulation of steroidogenesis and proliferation.

In the testis, two populations of Leydig cells, foetal Leydig cells (FLCs) and adult Leydig cells (ALCs), arise during prenatal and postnatal development, respectively. The differentiation and function of the both populations are regulated by autocrine/paracrine factors and endocrine hormones^1^. In the mouse, the FLC population arises at approximately 12.5 days postcoitum and is essential for masculinisation of the fetus^2^. The ALC population begins to arise at 4 days postpartum^3^. The development of the ALC population consists of three steps. First, spindle-shaped stem Leydig cells (SLCs) differentiate into spindle-shaped progenitor Leydig cells (PLCs), which express 3β-hydroxysteroid dehydrogenase (HSD3B) and luteinizing hormone/choriogonadotropin receptor (LHCGR). Second, the spindle-shaped PLCs transform into round-shaped immature Leydig cells (ILCs), which display high levels of androgen-metabolizing enzyme activity. Third, the ILCs mature into ALCs, and this maturation is accompanied by a further increase in cell size^4^,^5^,^6^,^7^. Although ALC development has long been studied predominantly in rat models, the detailed features of ALC development in a mouse model are not well characterized.

Oestrogen acts as an important regulator of cell proliferation, survival, and differentiation in a variety of organs and tissues. Oestrogen is present in both males and females. In particular, oestrogen regulates testicular function by promoting spermatogonial stem cell division and germ cell survival^8^,^9^,^10^. During early postnatal development in the rat, Sertoli cells are the primary source of testicular oestrogens^11^. In the rat, the testicular oestrogen levels decline when SLCs begin to differentiate into PLCs and then early ALCs; subsequently, these levels increase upon the emergence of mature ALCs^12^. Oestrogen has been found to inhibit the development of FLCs and ALCs. In organ cultures, oestrogens decrease the number of FLCs in foetal rat testes^13^. Exposure of mice to diethylstilbestrol (DES) *in utero* results in the accumulation of Leydig cells displaying an immature morphology^14^. Oestrogen treatment blocks the proliferation of PLCs isolated from immature rats and inhibits thymidine incorporation into ALCs isolated from adult rats^15^,^16^. In addition, oestrogen exerts an inhibitory effect on Leydig cell steroidogenesis. In the rat, maternal exposure to DES and 4-octylphenol results in a reduction in 17α-hydroxylase (CYP17A1) expression in the foetal testis^17^. Neonatal exposure to exogenous oestrogens until puberty reduces Leydig cell steroidogenesis in the rat^18^,^19^. Taken together, these findings indicate that oestrogen participates in Leydig cell development and in the maintenance of a stable Leydig cell population by regulating PLC and ALC proliferation.

The two oestrogen receptor isoforms, ESR1 and ESR2, are known to mediate the genomic action of oestrogen. Both oestrogen receptor subtypes are present in male reproductive organs^20^. Among these, ESR1 is expressed in the testes of fish, reptiles, birds, and mammals^21^,^22^,^23^,^24^,^25^,^26^,^27^,^28^,^29^, but the cellular specificity of ESR1 expression in the testis differs between species. In the rat and the marmoset monkey, ESR1 is expressed in Leydig cells from the late foetal stage through adulthood^25^. In the mouse, ESR1 is detected in Leydig cells of the adult testis^26^. Importantly, in adult male *Esr1* knockout mice, the testosterone (T) levels are increased, accompanied by an increase in the size of Leydig cells, which is associated with elevated expression of steroidogenic genes^30^,^31^,^32^. In addition, in adult rat Leydig cells, endogenous oestrogen inhibits the activities of steroidogenic enzymes via ESR1 action^33^. These suggest the participation of ESR1 in ALC function. However, at present, neither the expression pattern of ESR1 throughout ALC development nor the role of oestrogen receptor signalling in PLC steroidogenesis and proliferation is well understood. In the present study, we investigated the expression of ESR1 during the postnatal development of Leydig cells and the role of ESR1 in steroidogenesis and proliferation of PLCs in the mouse testes.

## Methods

### Experimental animals

Male ICR mice were purchased from Daehan Biolink (Eumseong, Korea). The testes were removed from the mice at postnatal day (PND) 1, 7, 14, 28, or 56 following asphyxiation using CO_2_. The date of birth was designated as PND 0. For the transgenic analysis, *Esr1* knockout mice in a C57BL6 background were produced as described previously^34^. Male littermates that expressed *Esr1* served as wild-type (WT) controls. The genotypes were confirmed by PCR of genomic DNA extracted from tail tips. WT and *Esr1* knockout mice at PND 14 were used. For PLC proliferation analysis, 17β-estradiol (E_2_) (Sigma-Aldrich, St. Louis, MO, USA), the ESR1-selective agonist propylpyrazoletriol (PPT; Abcam, Cambridge, UK), and the ESR2-selective agonist diarylpropionitrile (DPN; Abcam) were dissolved in DMSO and PBS (1:9, v/v). The male ICR mice received E_2_ (50 ng/g bw), PPT (100 ng/g bw), DPN (100 ng/g bw), or vehicle (100 μl) via ip injection every 2 days from PND 7 to PND 13. The mice were sacrificed at PND 14. At 3 hours before sampling, the mice were given an ip injection of bromodeoxyuridine (BrdU; 50 μg/g bw; Sigma-Aldrich). All animal procedures were approved by the Institutional Animal Care and Use Committee of Hanyang University (IACUC No. 15–0056 and 16–0062), and all experiments were performed in accordance with the Guide for the Care and Use of Laboratory Animals at Hanyang University.

### Isolation of seminiferous tubules and interstitial cells

Seminiferous tubules and interstitial cells were mechanically isolated as previously described^35^. Briefly, the testes were removed at PND 7, 14, 28, or 56 and were decapsulated in a culture dish containing DMEM/F12 medium. Under a dissecting microscope, interstitial cells and seminiferous tubules were separated using fine forceps. The isolated interstitial cells and seminiferous tubules were subjected to RT-PCR and Western blot analysis.

### Primary culture of Leydig cells

PLCs or ALCs were isolated from mice on PND 14 and 56, respectively. The testes were dissected, decapsulated in RPMI 1640 medium, and dispersed in conical tubes containing 0.5 mg/ml collagenase (type IV, Sigma-Aldrich) in a shaking incubator at 37 °C for 15 min. The tubes were briefly held in place, and then, the supernatant was collected. The Leydig cells were purified using a discontinuous Percoll density gradient (36% and 60% in PBS). The Percoll gradient was centrifuged at 800 × g for 30 min. The interface between the 36% and 60% phases was collected. PLCs and ALCs were cultured in RPMI 1640 medium containing 10% FBS at 37 °C for 24 hours. After starvation for 12 hours in serum-free medium, cells were treated with human chorionic gonadotropin (hCG; 0.5 IU/ml; Sigma-Aldrich), E_2_ (10 nM), PPT (20 nM), and DPN (20 nM). The purity of the Leydig cells was estimated based on HSD3B immunocytochemistry, and the percentage of PLCs and ALCs was greater than 80% and 90%, respectively.

### RT-PCR

Total RNA was isolated using Tri Reagent (Molecular Research Center, Cincinnati, OH, USA) and used for RT in ReverTra Ace qPCR RT master mix (Toyobo, Osaka, Japan). Real-time PCR was performed using AccuPower Greenstar qPCR master mix including SYBR (Bioneer, Daejeon, Korea) in a StepOnePlus real-time PCR system (Applied Biosystems, Foster City, CA, USA). Real-time PCR was performed using the following protocol: 5 min at 95 °C, 45 cycles of denaturation (10 sec at 95 °C) and annealing/extension (30 sec at 60 °C), and a final step of melting curve analysis. StepOne software (version 2.3, Applied Biosystems) was used to collect the PCR data. As an internal control, *ribosomal protein L7 (Rpl7*) was used. The relative level of mRNA was calculated using the 2^−ΔΔCt^ method. The primers are listed in Supplementary Table 1.

### Western blot analysis

Testes and isolated interstitial cells were homogenized and lysed in RIPA buffer (25 mM Tris-HCl at pH 7.6, 150 mM NaCl, 1% NP-40, 1% sodium deoxycholate, and 0.1% SDS) containing a protease inhibitor cocktail (Roche, Mannheim, Germany). Proteins were separated via 10% SDS-PAGE and transferred to a nitrocellulose membrane (Amersham Biosciences, Buckinghamshire, UK). The membrane was blocked using TBS containing 8% skim milk for 1.5 hours at room temperature. After rinsing with TBS containing 0.1% Tween-20 (TBST), the membrane was incubated in rabbit polyclonal anti-ESR1 antibody (1:1000; sc-542, Santa Cruz Biotechnology, Santa Cruz, CA, USA) and goat polyclonal anti-HSD3B antibody (1:1000; sc-30820, Santa Cruz Biotechnology) in 5% skim milk in TBS overnight at 4 °C. As an internal control, rabbit polyclonal anti-TUBB antibody (1:1000; sc-9104, Santa Cruz Biotechnology) was applied. After rinsing with TBST, the membrane was incubated in peroxidase-labeled goat anti-rabbit IgG (1:2000; ab6721, Abcam) or rabbit anti-goat IgG (1:2000; ab6741, Abcam) in 5% skim milk for 1 hour. The signal was detected using an ECL kit (Amersham Biosciences).

### Immunohistochemistry

Testes were fixed in Bouin’s solution (Sigma-Aldrich) overnight and then embedded in paraffin. Sections (5 μm) were mounted on poly-L-lysine-coated glass slides, deparaffinized, and rehydrated. Antigen retrieval was performed using 10 mM citrate buffer (pH 6.0) at 120 °C for 10 min. The slides were incubated for 10 min in 3% H_2_O_2_ in methanol to block endogenous peroxidase activity. Then, the samples were blocked using 5% goat serum for 30 min. The slides were incubated in rabbit polyclonal anti-ESR1 antibody (1:200) and goat polyclonal anti-HSD3B antibody (1:500) overnight at 4 °C. Normal rabbit IgG and goat IgG were used as negative controls, and antigen absorption tests were performed to determine the specificity of the polyclonal antibodies (Supplementary Fig. S1). After washing in PBS, the slides were incubated in peroxidase-labeled goat anti-rabbit IgG (1:200) or rabbit anti-goat IgG (1:500) for 30 min. After washing in PBS, the signal was developed using a 3,3′-diaminobenzidine (DAB) substrate kit (SK-4105, Vector Laboratories, Burlingame, CA, USA), and nuclei were stained using Harris hematoxylin. Permanently mounted slides were observed and photographed using a microscope (IX71, Olympus, Tokyo, Japan) equipped with a digital imaging system (DP71, Olympus).

### Quantitative image analysis

Quantitative image analysis was conducted using an image analysis program (iSolution Lite, ver. 7.8, IMT i-Solution, Vancouver, Canada) (Supplementary Fig. S2). ESR1 immunoreactivity in Leydig cells was measured, and the mean intensity of ESR1 staining was calculated. The numbers of each cell type were counted following immunostaining for HSD3B based on cell morphology as previously described^4^,^36^. Briefly, the numbers of HSD3B-positive round, HSD3B-negative spindle-shaped, and HSD3B-positive spindle-shaped cells were counted.

### Immunofluorescence double-labelling

Testes were fixed in 4% paraformaldehyde solution (Sigma-Aldrich) overnight at 4 °C and then embedded in paraffin. Sections (5 μm) were mounted on poly-L-lysine-coated glass slides, deparaffinized, and rehydrated. Antigen retrieval was performed using 10 mM citrate buffer (pH 6.0) at 120 °C for 10 min. The sections were blocked in 5% donkey serum for 30 min and incubated in rabbit polyclonal anti-ESR1 antibody (1:50) and goat polyclonal anti-HSD3B antibody (1:50) overnight at 4 °C. After washing in PBS, the sections were incubated in Alexa Fluor 568-conjugated donkey anti-rabbit IgG (1:200; ab175692, Abcam) and Alexa Fluor 488-conjugated donkey anti-goat IgG (1:200; ab150133, Abcam) for 1 hour. After washing with PBS, the sections were mounted using ProLong Gold Antifade Reagent with DAPI (Invitrogen, Eugene, OR, USA) and were observed under an epifluorescence microscope equipped with a digital imaging system. For BrdU staining, rat monoclonal anti-BrdU antibody (1:100; ab6326, Abcam) and Alexa Fluor 568-conjugated donkey anti-rat IgG (1:200; ab175475, Abcam) were applied. The numbers of BrdU-positive/HSD3B-positive and BrdU-negative/HSD3B-positive cells were estimated. More than 600 cells per testis were counted.

Leydig cell smears or cultured Leydig cells were fixed in ice-cold methanol for 10 min, permeabilized in 0.2% Triton X-100 for 10 min, and then blocked in 5% donkey serum for 30 min. The cells were incubated overnight at 4 °C in rabbit polyclonal anti-ESR1 antibody (1:50), goat polyclonal anti-HSD3B antibody (1:50), and rabbit polyclonal anti-KI67 antibody (1:100; ab66155, Abcam). After washing in PBS, the cells were incubated in Alexa Fluor 568-conjugated donkey anti-rabbit IgG (1:200) and Alexa Fluor 488-conjugated donkey anti-goat IgG (1:200) for 1 hour. After washing in PBS, the cells were mounted using ProLong Gold Antifade Reagent with DAPI. The numbers of ESR1-positive/HSD3B-positive cells and ESR1-positive/HSD3B-negative cells were estimated in Leydig cell smears. In cultured Leydig cells, the numbers of HSD3B-positive/KI67-positive and HSD3B-positive/KI67-negative cells were estimated. More than 300 cells per condition were counted.

### Hormone assay

The concentrations of progesterone (P_4_) and T in the spent media were measured via electrochemiluminescence immunoassay (ECLIA). The assay procedure was performed using a Modular Analytics E170 analyzer (Elecsys, Roche Diagnostics, Indianapolis, IN, USA) according to manufacturer’s protocol. The P_4_ and T levels were expressed as ng/ml per 10^6^ cells.

### Statistical analysis

Statistical significance was analyzed using Student’s *t*-test or one-way analysis of variance (ANOVA) followed by Tukey’s test using SPSS software (Chicago, IL, USA). Significance was accepted when the *P*-value was smaller than 0.05.

## Results

### ESR1 is differentially expressed in the mouse testes during postnatal development

**Figure 1:**
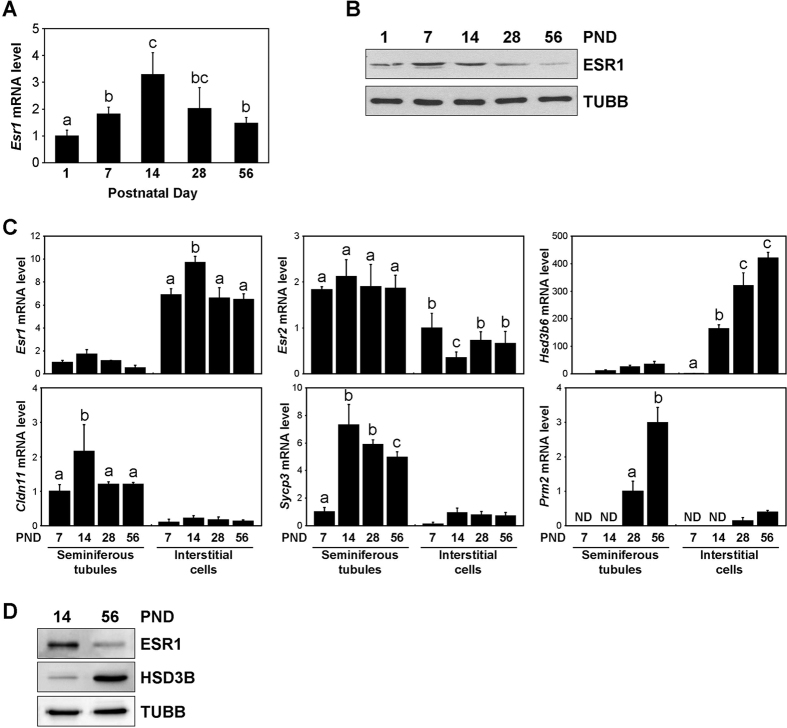
Expression of *Esr1* in the mouse testes during postnatal development. Real-time PCR (**A**) and Western blot analysis (**B**) of *Esr1* expression in the testes at PND 1, 7, 14, 28, and 56. As internal controls, *Rpl7* mRNA and TUBB protein were assessed, respectively. The data are presented as the means + SD (n = 4). Different letters indicate significant differences (*P* < 0.05). (**C**) Real-time RT-PCR of *Esr1* and *Esr2* mRNA expression in the seminiferous tubules and interstitial cells at PND 7, 14, 28, and 56. *Hsd3b6* mRNA was examined as a Leydig cell marker. *Cldn11, Sycp3,* and *Prm2* mRNAs were examined as markers of Sertoli cells, spermatocytes, and spermatids, respectively. *Rpl7* mRNA was used as an internal control. The data are presented as the means + SD (n = 4). Different letters indicate significant differences (*P* < 0.05). ND, not detected. (**D**) Western blot analysis of ESR1 and HSD3B protein expression in the interstitial cells at PND 14 and 56. TUBB protein was used as an internal control.

*Esr1* mRNA and protein expression in the mouse testes was examined at PND 1, 7, 14, 28, and 56. The *Esr1* mRNA levels significantly increased from PND 1 to 14 and decreased thereafter until PND 56 (Fig. 1A). Based on Western blot, the ESR1 protein level increased in the testes from PND 1 to 14 and decreased thereafter until PND 56 (Fig. 1B). The highest ESR1 protein level was observed at PND 7. To verify the tissue-specific expression of *Esr1* mRNA in the developing testis, real-time RT-PCR was performed on seminiferous tubules and interstitial cells isolated from mouse testes at PND 7, 14, 28, and 56 (Fig. 1C). In both seminiferous tubules and interstitial cells, the *Esr1* mRNA level increased from PND 7 to 14 and decreased thereafter. This result indicated that the observation of maximal ESR1 protein expression in the testis at PND 7 is due to the presence of fewer seminiferous tubular cells relative to PND 14. The *Esr2* mRNA level in the seminiferous tubules was constant from PND 7 to 56. In the interstitial cells, the *Esr2* mRNA level was the lowest at PND 14. The *Hsd3b type 6 (Hsd3b6*) mRNA level increased gradually from PND 7 to 56 in the interstitial cells. To determine the purity of the isolated interstitial cells, *claudin-11 (Cldn11*), *synaptonemal complex protein-3 (Sycp3*), and *protamine-2 (Prm2*), markers of Sertoli cells, spermatocytes, and spermatids, respectively, were examined. Low levels of *Cldn11, Sycp3*, and *Prm2* mRNA were detected in the interstitial cells. On Western blot, the ESR1 protein level decreased in the interstitial cells from PND 14 to 56, whereas the HSD3B protein level increased (Fig. 1D).

### HSD3B-positive PLCs strongly express ESR1

**Figure 2:**
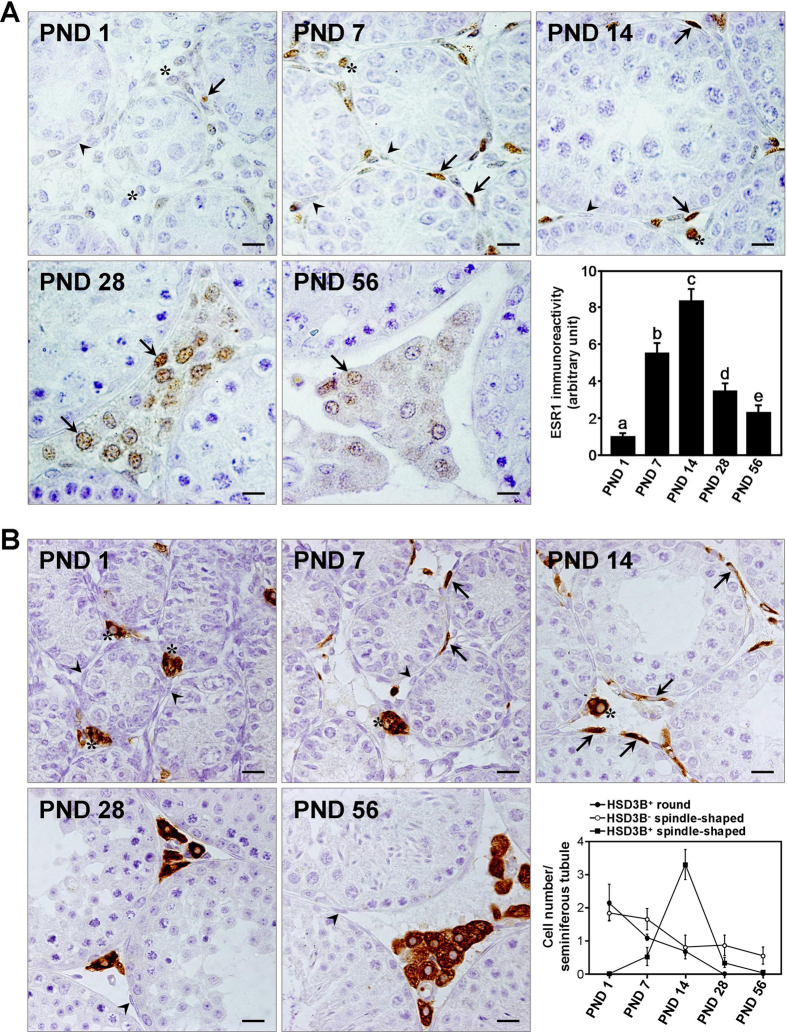
Immunohistochemical analysis of ESR1 and HSD3B expression in the mouse testes during postnatal development. (**A**) Immunostaining for ESR1 in the testes at PND 1, 7, 14, 28, and 56. Asterisks indicate FLCs. Arrows indicate ESR1-positive cells, and arrowheads denote ESR1-negative spindle-shaped cells. *Scale bars,* 10 μm. *Lower right*, quantitative image analysis of nuclear ESR1 immunoreactivity in Leydig cells. The data are presented as the means + SD (n = 4). Different letters indicate significant differences (*P* < 0.05). (**B**) Immunostaining for HSD3B in the testes at PND 1, 7, 14, 28, and 56. Asterisks indicate HSD3B-positive round cells, presumed to be FLCs; arrowheads denote HSD3B-negative spindle-shaped cells; and arrows denote HSD3B-positive spindle-shaped cells, presumed to be PLCs. *Scale bars,* 10 μm. *Lower right*, number of HSD3B-positive round, -negative spindle-shaped, and -positive spindle-shaped cells per seminiferous tubule. The data are presented as the means ±SD (n = 4).

To investigate the ESR1 expression in developing Leydig cells, we performed immunohistochemistry on mouse testicular tissue at PND 1, 7, 14, 28, and 56. In the developing testis, ESR1 immunoreactivity was observed in interstitial cells but not in germ cells or Sertoli cells (Fig. 2A). In the interstitial cells, ESR1 immunoreactivity was detected in nuclei. In particular, hexagonal FLCs in the interstitium expressed ESR1 negligibly at PND 1 but strongly from PND 7 to 14. At PND 7 and 14, some of spindle-shaped cells showed strong immunoreactivity for ESR1. At PND 28 and 56, ESR1 immunoreactivity in polygonal Leydig cells was decreased. Quantitative image analysis revealed that nuclear ESR1 immunoreactivity increased by 5.5- and 8.3-fold at PND 7 and 14, respectively, relative to that at PND 1. Thereafter, nuclear ESR1 immunoreactivity decreased by 2.4- and 3.6-fold at PND 28 and 56, respectively, relative to that at PND 14. To track the Leydig cell lineage in the developing testis, immunohistochemistry for HSD3B was performed on the mouse testes (Fig. 2B). At PND 1, HSD3B immunoreactivity was detected in hexagonal cells, presumed to be FLCs, located in the interstitium but not in spindle-shaped cells in the periphery of the seminiferous tubule. At PND 7, HSD3B-positive spindle-shaped cells located in the periphery of seminiferous tubule and interstitium, presumed to be PLCs, appeared together with HSD3B-negative spindle-shaped cells. At PND 14, HSD3B-positive spindle-shaped cells were abundant but HSD3B-negative spindle-shaped cells and FLCs were rarely found among the interstitial cell population. At PND 28, polygonal HSD3B-positive cells, presumed to be ILCs, were abundant in the interstitium, but HSD3B-positive spindle-shaped cells were rare. At PND 56, round HSD3B-positive cells with an increased size, presumed to be ALCs, were predominantly found. Quantitative analysis demonstrated that the number of PLCs peaked at PND 14.

**Figure 3:**
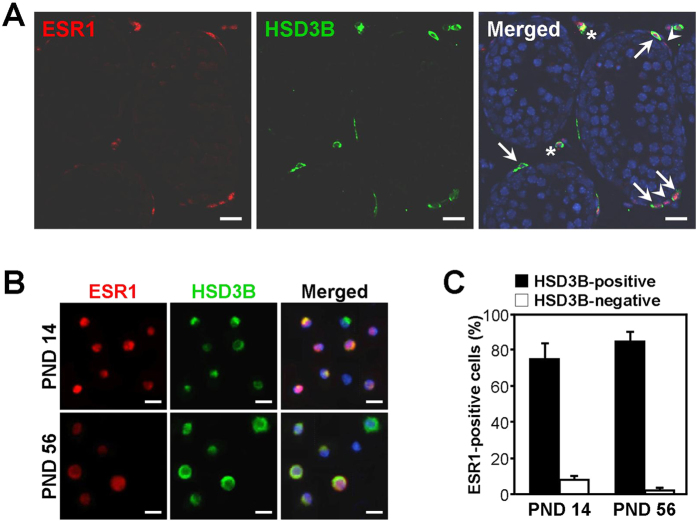
Expression of ESR1 and HSD3B in mouse Leydig cells. (**A**) Immunofluorescence double-labelling for ESR1 (red) and HSD3B (green) in the testis at PND 14. DAPI staining (blue) was performed to label nuclei. Asterisks indicate FLCs. Arrows denote ESR1-positive/HSD3B-positive cells, and arrowheads denote ESR1-positive/HSD3B-negative cells. *Scale bars,* 20 μm. (**B**) Leydig cells freshly isolated from the testes at PND 14 and 56 were double-stained with ESR1 and HSD3B antibodies. *Scale bars,* 10 μm. (**C**) The percentages of ESR1-positive cells that were positive or negative for HSD3B among the isolated Leydig cells. The data are presented as the means + SD (n = 4).

To verify the expression of ESR1 in PLCs, the mouse testis at PND 14 was subjected to immunofluorescence double-labelling for ESR1 and HSD3B (Fig. 3A). ESR1-positive/HSD3B-positive cells were found in the periphery of seminiferous tubules. ESR1-positive/HSD3B-negative cells were also found. Leydig cells isolated from mouse testes at PND 14 and 56 were double-labeled with ESR1 and HSD3B antibodies (Fig. 3B). At PND 14, 75% of HSD3B-positive and 8% of HSD3B-negative cells were ESR1-positive (Fig. 3C). At PND 56, 85% of HSD3B-positive and 2% of HSD3B-negative cells were ESR1-positive.

### E_2_ and PPT suppress hCG-induced steroidogenesis in PLCs and ALCs

**Figure 4:**
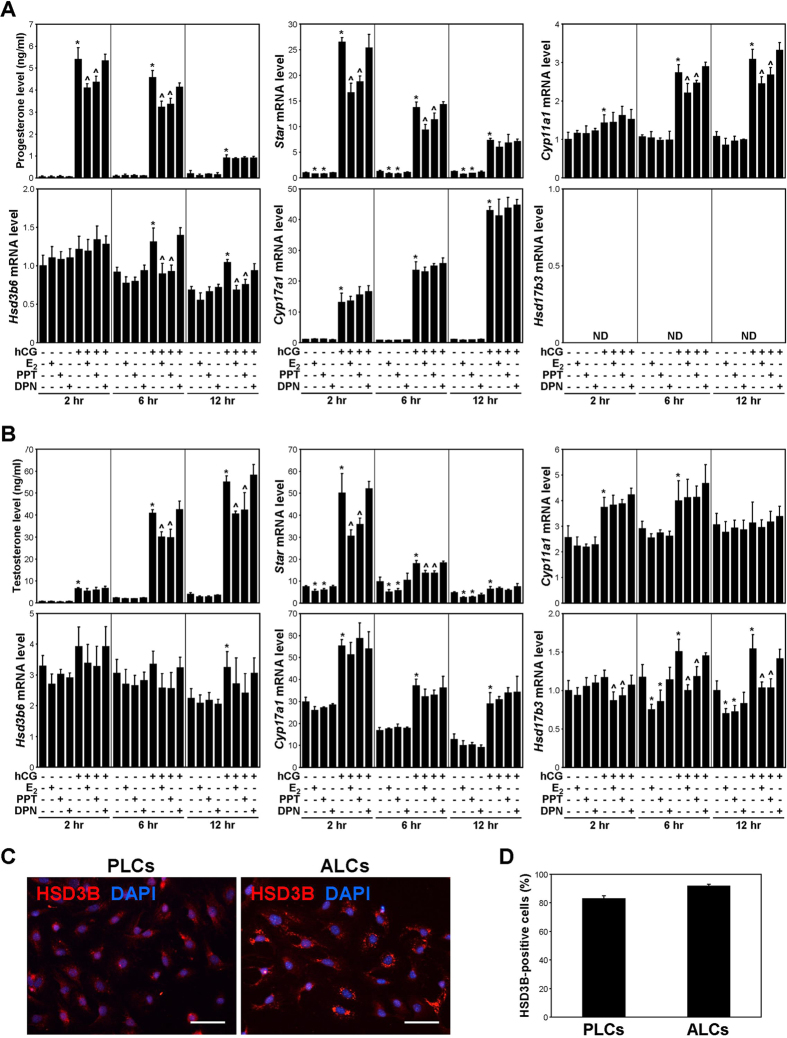
Effects of E_2_, PPT, and DPN on steroidogenesis by PLCs and ALCs. PLCs (**A**) and ALCs (**B**) were treated with E_2_, PPT, or DPN in combination with hCG for 2, 6, or 12 hours. The P_4_ levels in the PLC culture medium and the T levels in the ALC culture medium were measured via ECLIA. The mRNA levels of steroidogenic genes in PLCs and ALCs were measured via real-time PCR. As an internal control, *Rpl7* mRNA was used. The data are presented as the means + SD (n = 4). ND, not detected. *P < 0.05, compared with the time-matched vehicle-treated control cells; ^^^*P* < 0.05, compared with the time-matched cells treated with hCG alone. (**C**) Immunolabelling for HSD3B (red) in the PLC and ALC culture. The cells were stained using a rabbit polyclonal anti-HSD3B antibody. DAPI staining (blue) was performed to label nuclei. *Scale bars,* 50 μm. (**D**) The percentages of HSD3B-positive cells in the PLC and ALC culture. The data are presented as the means + SD (n = 4).

To determine whether the effect of oestrogen on the steroidogenesis of PLCs is mediated by ESR1, Leydig cells (primarily PLCs) isolated at PND 14 were treated with E_2_, PPT, and DPN in combination with hCG (Fig. 4A). In cultured PLCs, the hCG-induced P_4_ levels in the spent medium were significantly decreased by E_2_ and PPT treatment, but not by DPN treatment, at 2 and 6 hours. The maximal P_4_ level was observed at 2 hours post-hCG treatment and declined thereafter until 12 hours. T was not detected in the PLC culture medium. The *steroidogenic acute regulatory protein (Star*), *cholesterol side-chain cleavage enzyme (Cyp11a1*), and *Hsd3b6* mRNA levels were significantly decreased by E_2_ and PPT but not by DPN. The *Cyp17a1* mRNA level was not changed by E_2_, PPT, or DPN. *17β-hydroxysteroid dehydrogenase type 3 (Hsd17b3*) mRNA was not detected in PLCs. In the ALC culture medium, the T levels were significantly decreased by E_2_ and PPT treatment but not by DPN treatment (Fig. 4B). The *Star* and *Hsd17b3* mRNA levels were significantly decreased by E_2_ and PPT but were not changed by DPN. The *Cyp11a1, Cyp17a1* and *Hsd3b6* mRNA levels were not changed by E_2_, PPT, or DPN.

To verify the purity of the Leydig cells, the PLCs (isolated at PND 14) and ALCs (isolated at PND 56) before any kind of treatment were estimated using HSD3B immunocytochemistry (Fig. 4C). The intensity of HSD3B immunofluorescence was moderate in the PLCs, and was strong in the ALCs. The percentages of HSD3B-positive cells were 82.8% and 91.7% in the PLC and ALC culture, respectively (Fig. 4D).

### E_2_ do not suppress hCG-induced steroidogenesis in PLCs from *Esr1* knockout mice

**Figure 5:**
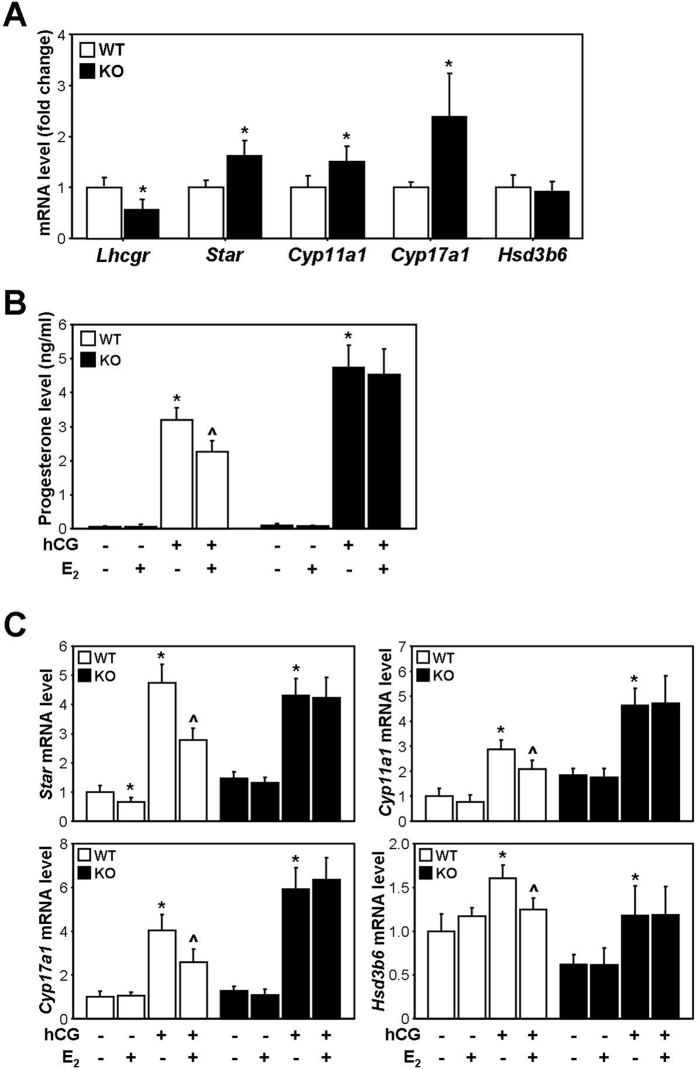
Effect of *Esr1* deletion on PLC steroidogenesis. (**A**) mRNA expression of steroidogenic genes in Leydig cells isolated from WT or *Esr1* knockout (KO) mice at PND 14. The data are presented as the means + SD (n = 4). *P < 0.05, compared with WT. (**B**,**C**) Effect of E_2_ on PLC steroidogenesis in *Esr1* knockout (KO) mice. Cells were treated with E_2_ in combination with hCG for 3 hours. The P_4_ levels in the culture medium were measured via ECLIA. The mRNA levels of steroidogenic genes were measured via real-time PCR. As an internal control, *Rpl7* mRNA was used. The data are presented as the means + SD (n = 4). *P < 0.05, compared with the genotype-matched vehicle-treated control cells; ^^^*P* < 0.05, compared with the genotype-matched cells treated with hCG alone.

To verify the effect of *Esr1* deletion on PLC-mediated steroidogenesis, PLCs isolated from *Esr1* knockout and WT mice at PND 14 were directly subjected to RT-PCR analysis or treated with E_2_ in combination with hCG for 3 hours. In isolated Leydig cells, the *Lhcgr* mRNA levels were significantly lower in *Esr1* knockout mice than in WT mice, whereas the *Star, Cyp11a1*, and *Cyp17a1* mRNA levels were significantly higher in *Esr1* knockout mice than in WT mice (Fig. 5A). In the PLC culture medium, E_2_ treatment decreased the hCG-induced P_4_ level in cells isolated from WT mice but not from *Esr1* knockout mice (Fig. 5B). The *Star, Cyp11a1*, and *Cyp17a1*, and *Hsd3b6* mRNA levels were significantly decreased by E_2_ in cells from WT mice but were not changed by E_2_ in cells from *Esr1* knockout mice (Fig. 5C).

### E_2_, PPT, and DPN inhibit PLC proliferation in neonatal mice

**Figure 6:**
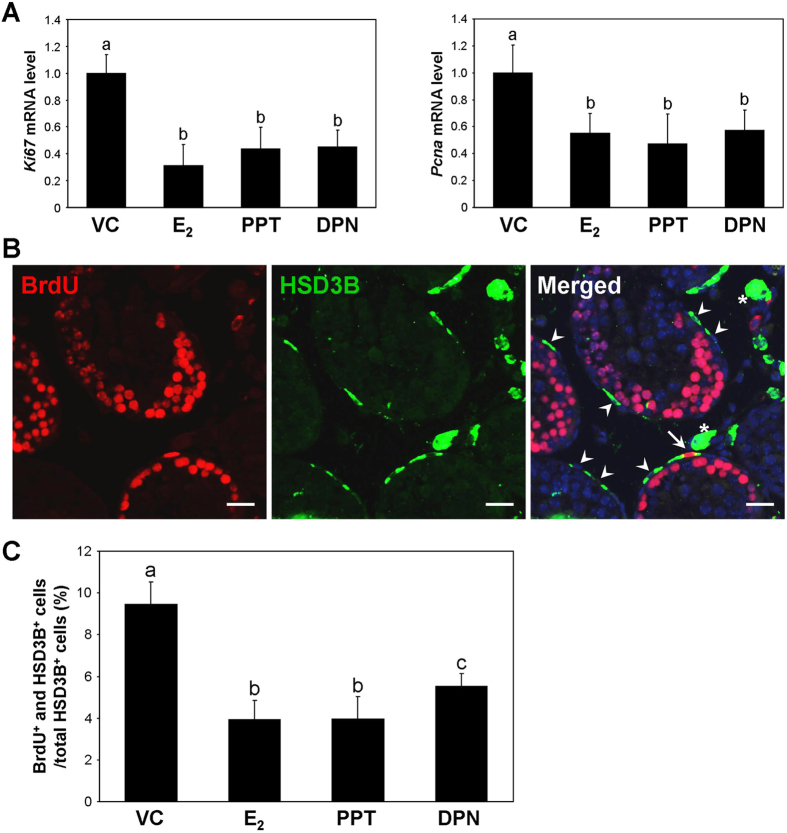
Effect of oestrogens on PLC proliferation in the neonatal mouse testes. (**A**) Effects of E_2_, PPT, and DPN on *Ki67* and *Pcna* mRNA expression in Leydig cells isolated from neonatal mice. The data are presented as the means + SD (n = 4). Different letters indicate significant differences (*P* < 0.05). (**B**) Immunofluorescence double-labelling for BrdU (red) and HSD3B (green). Asterisks indicate FLCs. The arrow denotes a BrdU-positive/HSD3B-positive cell, and arrowheads denote BrdU-negative/HSD3B-positive cells. *Scale bars*, 20 μm. (**C**) Percentages of cells double-positive for BrdU and HSD3B. The data are presented as the means + SD (n = 4). Different letters indicate significant differences (*P* < 0.05).

Neonatal male mice received E_2_, PPT, or DPN and were administered BrdU before they were sacrificed. Testis samples and Leydig cells isolated from these mice were subjected to proliferation analysis. In Leydig cells, the mRNA levels of the cell proliferation markers, *Ki67* and *proliferating cell nuclear antigen (Pcna*), were decreased by E_2_, PPT, and DPN treatment (Fig. 6A). To determine whether PLC proliferation was affected by oestrogen treatment, immunofluorescence double-labelling for HSD3B and BrdU was conducted, and the percentages of HSD3B-positive and BrdU-positive cells were determined (Fig. 6B,C). The percentages of HSD3B-positive and BrdU-positive cells were decreased by E_2_, PPT, and DPN treatment.

### E_2_, PPT, and DPN suppress hCG-induced PLC proliferation

**Figure 7:**
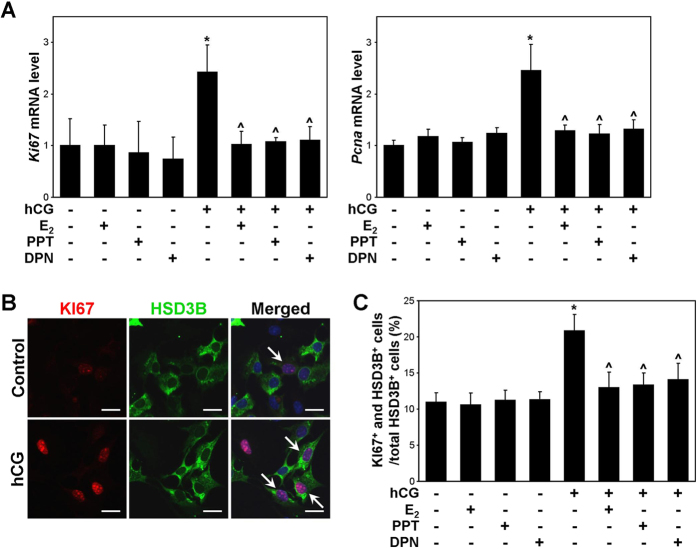
Effect of oestrogens on PLC proliferation in primary culture. PLCs were treated with E_2_, PPT, or DPN in combination with hCG for 24 hours. (**A**) The *Ki67* and *Pcna* mRNA levels were measured via real-time PCR. *Rpl7* mRNA was used as an internal control. The data are presented as the means + SD (n = 4). *P < 0.05, compared with the vehicle-treated control cells; ^^^*P* < 0.05, compared with the cells treated with hCG alone. (**B**) The cells were double-immunostained with anti-HSD3B and anti-KI67 antibodies. *Scale bars,* 20 μm. (**C**) The percentages of cells double-positive for HSD3B and KI67 (arrows) were calculated. The data are presented as the means + SD (n = 4). *P < 0.05, compared with the vehicle-treated control cells; ^^^*P* < 0.05, compared with the cells treated with hCG alone.

To examine the role of oestrogen-oestrogen receptor signalling in the proliferation of PLCs, the mRNA expression of *Ki67* and *Pcna* was examined in isolated PLCs following hCG, E_2_, PPT, and DPN treatment for 24 hours. The mRNA levels of *Ki67* and *Pcna* were significantly increased by hCG treatment, and this increase was attenuated by E_2_, PPT, and DPN treatment (Fig. 7A). However, in non-hCG-treated PLCs, treatment with E_2_, PPT, or DPN did not change the *Ki67* or *Pcna* mRNA levels. Based on immunocytochemical double-labelling analysis, the percentages of HSD3B-positive and KI67-positive cells were significantly increased by hCG treatment, and this increase was attenuated by E_2_, PPT, and DPN treatment (Fig. 7B,C). However, in non-hCG-treated PLCs, treatment with E_2_, PPT, or DPN did not change the number of HSD3B-positive or KI67-positive cells.

## Discussion

In the mouse and rat testis, the FLC population arises from stem cells present in the foetal gonads, accumulates during foetal development, and declines after birth^37^,^38^. In human FLCs, LHCGR is bound to placental hCG during the first stage and by pituitary LH during the last stage of gestation^39^,^40^. In rodents, the initial development and maintenance of FLCs do not require LH, but androgen synthesis by FLCs gradually becomes LH-dependent immediately before birth^2^. The androgen synthesis peaks just prior to birth and gradually decreases from birth to prepuberty^41^. The present study showed that ESR1 immunoreactivity in FLCs was low at PND 1 and increased thereafter until PND 14 (Fig. 2A). In male *Esr1* knockout mice, the testicular T levels and the mRNA levels of *Star, Cyp11a1*, and *Cyp17a1* are higher than those in WT mice at gestational day 13.5 and PND 2^42^. In the rat, neonatal exposure to oestrogens until prepuberty results in reduced T levels together with abnormal testis development^18^,^19^. Taken together, these findings indicate that oestrogen-ESR1 signalling may inhibit FLC-mediated steroidogenesis.

ESR1 is expressed in Leydig cells of the adult mouse testis^26^, but the temporal changes in ESR1 expression during ALC development have remained unexplored. Quantitative analysis of HSD3B expression during the postnatal development of the Leydig cells revealed that the PLC population increased from PND 1 to 14 and decreased thereafter (Fig. 2B). Alternatively, the populations of HSD3B-positive round cells (FLCs) and 3βHSD-negative spindle-shaped cells, of which some are SLCs, decreased from PND 1 to 14. ESR1 immunoreactivity revealed that ESR1 expression in Leydig cells increased from PND 1 to 14 and gradually decreased thereafter (Fig. 2A). Concomitantly, *Esr1* mRNA expression was increased in interstitial cells between PND 7 and 14 and decreased thereafter (Fig. 1C). *Esr1* mRNA expression was also detected in the seminiferous tubules at PND 7 and 14. This result might be attributable to the attachment of ESR1-expressing spindle-shaped cells to the seminiferous tubules. It would be more helpful if *in situ* hybridization data were provided which cell types express the *Esr1* mRNA in mouse testes. Based on double-immunolabelling for ESR1 and HSD3B in Leydig cells isolated from mouse testis at PND 14, most cells were positive for both HSD3B and ESR1 (Fig. 3). Taking into account that PLCs were predominantly abundant in the interstitium but that the FLC population was barely detectable at PND 14 in the mouse testis, the highest level of ESR1 in the interstitium at PND 14 suggests an intriguing role of ESR1 in PLCs. In addition, these data demonstrate that PLCs exhibit higher level of ESR1 than do ALCs in the mouse, which is similar with that of rat model^24^. In the rat, PLCs are absent at PND 7^36^. Alternatively, the present study showed that PLCs are present at PND 7, indicating the difference of Leydig cell development between rat and mouse.

The predominant androgen produced by PLCs and ALCs is androsterone and T, respectively^6^. Although androsterone was not measured in the spent cell culture media, the level of P_4_, which is converted to androgens, was decreased by E_2_ and PPT in hCG-stimulated PLCs (Fig. 4A). Taking into account that the *Star, Cyp11a1*, and *Hsd3b6* mRNA levels were decreased by E_2_ and PPT, oestrogen-ESR1 signalling may inhibit androgen synthesis in hCG-stimulated PLCs at the levels of P_4_ synthesis and of androsterone synthesis. Notably, in cultured PLCs, DPN treatment did not change the mRNA levels of steroidogenic genes or the level of P_4_ secretion. Furthermore, E_2_ did not suppress hCG-induced steroidogenesis in PLCs isolated from *Esr1* knockout mice (Fig. 5B,C). These results suggest that oestrogen inhibits PLC-mediated steroidogenesis via ESR1 but not ESR2. Although the mRNA expression of *Star* was decreased by E_2_ and PPT in PLCs in the absence of hCG, the basal level of androgen synthesis was not significantly altered. This result suggests that the inhibitory effect of oestrogen-ESR1 signalling on androgen synthesis in PLCs may be more potent during LH stimulation. It is well known that oestrogens inhibit steroidogenesis by mature ALCs. In cultured ALCs, the level of T in the culture medium and the mRNA levels of steroidogenic pathway genes were down-regulated by E_2_ and PPT upon stimulation with hCG (Fig. 4B), and this result is consistent with those of previous studies^30^,^43^,^44^. Taken together, these findings indicate that oestrogens may inhibit steroidogenesis primarily via ESR1 in the ALC lineage.

Oestrogens have been known to inhibit the proliferation of Leydig cells, and this effect appears to be dependent on the stage of the Leydig cell lineage^12^. Among the cell types of the ALC lineage, PLCs are more actively proliferative than ILCs and ALCs^16^,^45^. In the rat, LH stimulates PLC proliferation, whereas oestrogen exerts an inhibitory effect on PLC proliferation^16^,^46^,^47^,^48^,^49^. In the present study, BrdU incorporation in PLCs of neonatal mice was decreased by E_2_, PPT, and DPN treatment, and this effect was accompanied by a decrease in the *Ki67* and *Pcna* mRNA levels (Fig. 6). In addition, in PLCs isolated at PND 14 and treated with E_2_, PPT, or DPN in combination with hCG, E_2_ and PPT abrogated the hCG-induced increases in the *Ki67* and *Pcna* mRNA levels and the KI67-positive cell number (Fig. 7). This result suggests that oestrogen negatively regulates the hCG-stimulated proliferation of PLCs via ESR1. Of note, DPN also significantly attenuated the hCG-induced increases in the *Ki67* and *Pcna* mRNA levels and the KI67-positive cell number. Taken together, these findings indicate that the inhibitory effect of oestrogen on hCG-induced PLC proliferation may be mediated by both ESR1 and ESR2. However, in rat PLCs, 50 nM E_2_ alone significantly reduces the proliferation of PLCs in the absence of hCG stimulation^16^. Taking into account that 10 nM E_2_ was applied to mouse PLCs in the present study, the different effect of E_2_ on PLC proliferation in the absence of hCG between the rat and mouse models might be attributable to tested dosage of E_2_.

In past decades, it has been claimed that exposure to environmental oestrogens is linked to the alteration of male reproductive function. Indeed, the frequency of male reproductive disorders, such as low sperm counts, testicular cancer, cryptorchidism, and hypospadia, has increased in humans during the past several decades^50^,^51^. In particular, endocrine disruption in Leydig cells occurs when animals are exposed to xenoestrogens during the neonatal and prepubertal periods^18^,^19^,^52^,^53^,^54^. Taking into account the high levels of ESR1 expression in PLCs, xenoestrogen-ESR1 signalling may deregulate normal PLC development and steroidogenesis during the neonatal and prepubertal periods. In ALCs in the adult testis, the ESR1 expression levels were lower than those in PLCs, suggesting that endocrine disruption via xenoestrogen-ESR1 signalling could be weaker than that observed in neonates.

**Figure 8:**
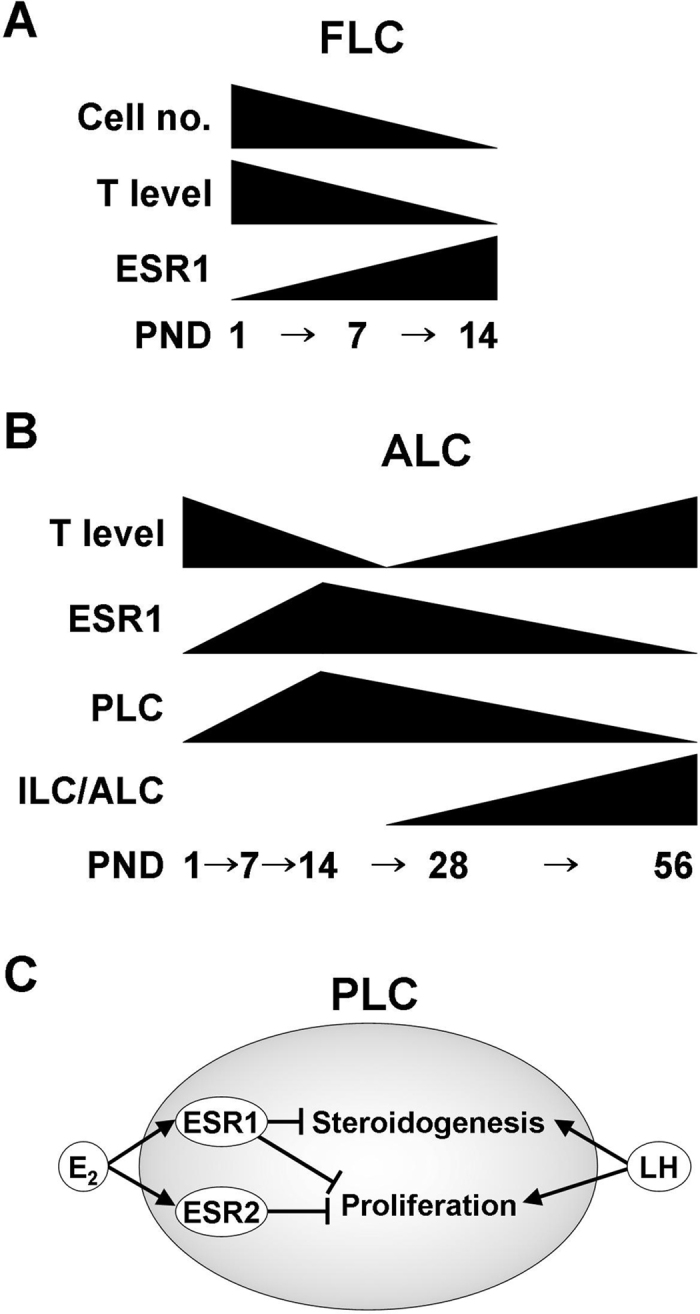
Schematic representation of ESR1 expression in Leydig cells. (**A**) The expression of ESR1 in mouse FLCs during early postnatal development. (**B**) The expression of ESR1 throughout ALC development from birth to adulthood. (**C**) Working model illustrating E_2_ function in PLCs. In PLCs, hCG-stimulated steroidogenesis is negatively regulated by oestrogen-ESR1 signalling, whereas the hCG-stimulated proliferation of PLCs is negatively regulated by oestrogen-ESR1 and oestrogen-ESR2 signalling.

ESR1 expression was increased in both FLCs and PLCs during neonatal development and was subsequently decreased during ALC development from PLCs, suggesting that the ESR1 level is a potential biomarker of Leydig cell development (Fig. 8A,B). In PLCs, hCG-stimulated steroidogenesis was negatively regulated by oestrogen-ESR1 signalling, whereas hCG-stimulated proliferation was negatively regulated by oestrogen-ESR1 and oestrogen-ESR2 signalling (Fig. 8C). The negative regulation of PLC steroidogenesis and proliferation by oestrogen-ESR1 signalling should be considered as a mechanism of the endocrine control of Leydig cell development in the testis.

## Additional Information

**How to cite this article:** Oh, Y. S. *et al*. ESR1 inhibits hCG-induced steroidogenesis and proliferation of progenitor Leydig cells in mice. *Sci. Rep.* **7**, 43459; doi: 10.1038/srep43459 (2017).

**Publisher's note:** Springer Nature remains neutral with regard to jurisdictional claims in published maps and institutional affiliations.

## Supplementary Material

Supplementary Figure and Table
