# Supplementary material for: ESR1 inhibits hCG-induced steroidogenesis and proliferation of progenitor Leydig cells in mice
Source: Sci Rep. 2017 Mar 7;7:43459. doi: 10.1038/srep43459 (PMC5339920; doi:10.1038/srep43459)
Supplement: Supplementary Figure and Table [file srep43459-s1.pdf]

ESR1 inhibits hCG-induced steroidogenesis and proliferation of progenitor Leydig cells in mice

Yeong Seok Oh, Il Kyoo Koh, Bomi Choi, and Myung Chan Gye*

Department of Life Science and Research Institute for Natural Sciences, Hanyang University, Seoul 04763, Korea

*Corresponding: mcgye@hanyang.ac.kr (M.C.G.)

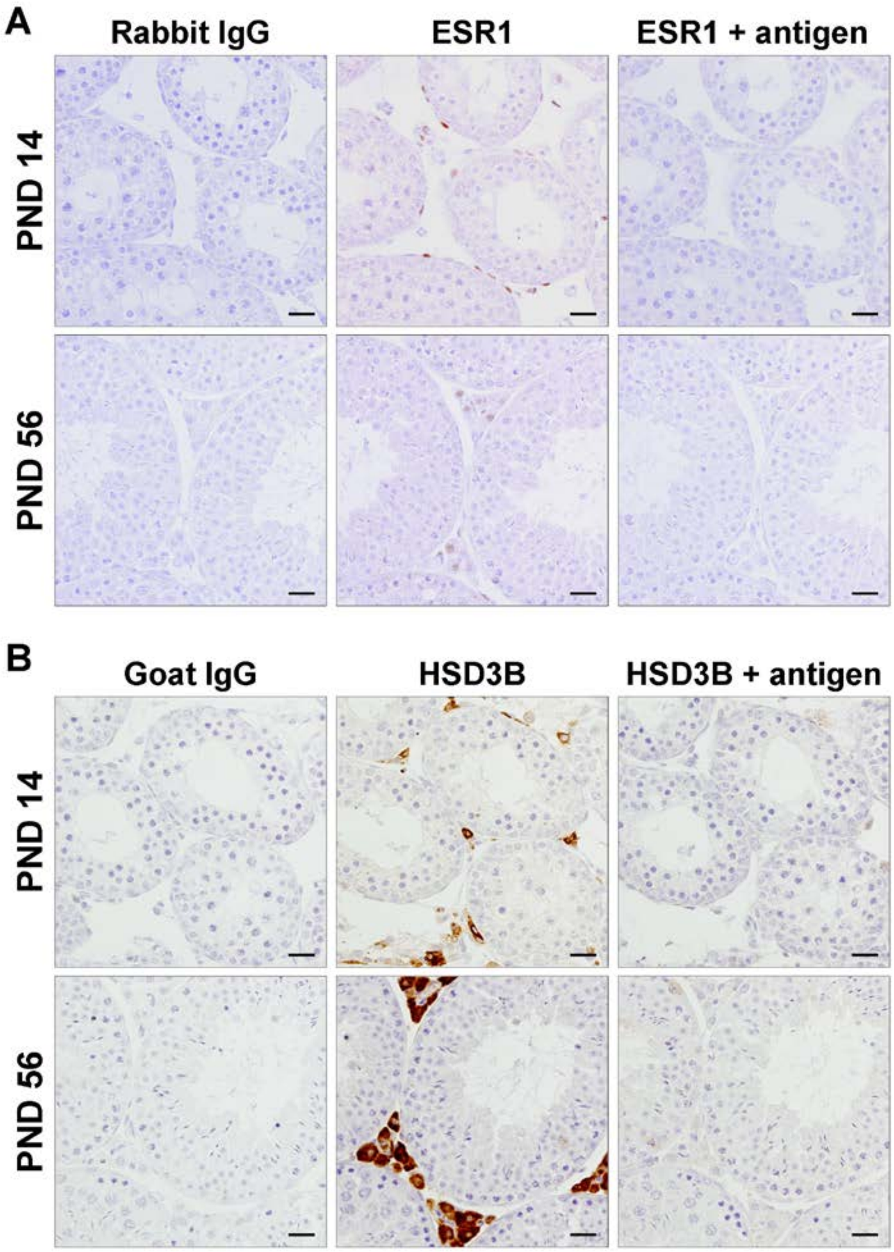

Supplementary Fig. S1. Specificity of antibodies on immunohistochemistry. (A) Immunostaining for ESR1 in mouse testes at PND 14 and 56. As a negative control, normal rabbit IgG (1 μg/ml, ab27478, Abcam) was used. Antigen peptide (5 μg/ml; sc-542 P, Santa Cruz Biotechnology) was incubated with rabbit polyclonal anti-ESR1 antibody (1 μg/ml, sc-542, Santa Cruz Biotechnology) for antigen absorption test. No specific signal was found in the negative control and the antigen absorption test. Scale bars, 20 μm. (B) Immunostaining for HSD3B in mouse testes at PND 14 and 56. As a negative control, normal goat IgG (0.4 μg/ml, sc-2028, Santa Cruz Biotechnology) was used. Antigen peptide (2 μg/ml; sc-30820 P, Santa Cruz Biotechnology) was incubated with goat polyclonal anti-HSD3B antibody (0.4 μg/ml, sc-30820, Santa Cruz Biotechnology) for antigen absorption test. No specific signal was found in the negative control and the antigen absorption test. Scale bars, 20 μm.

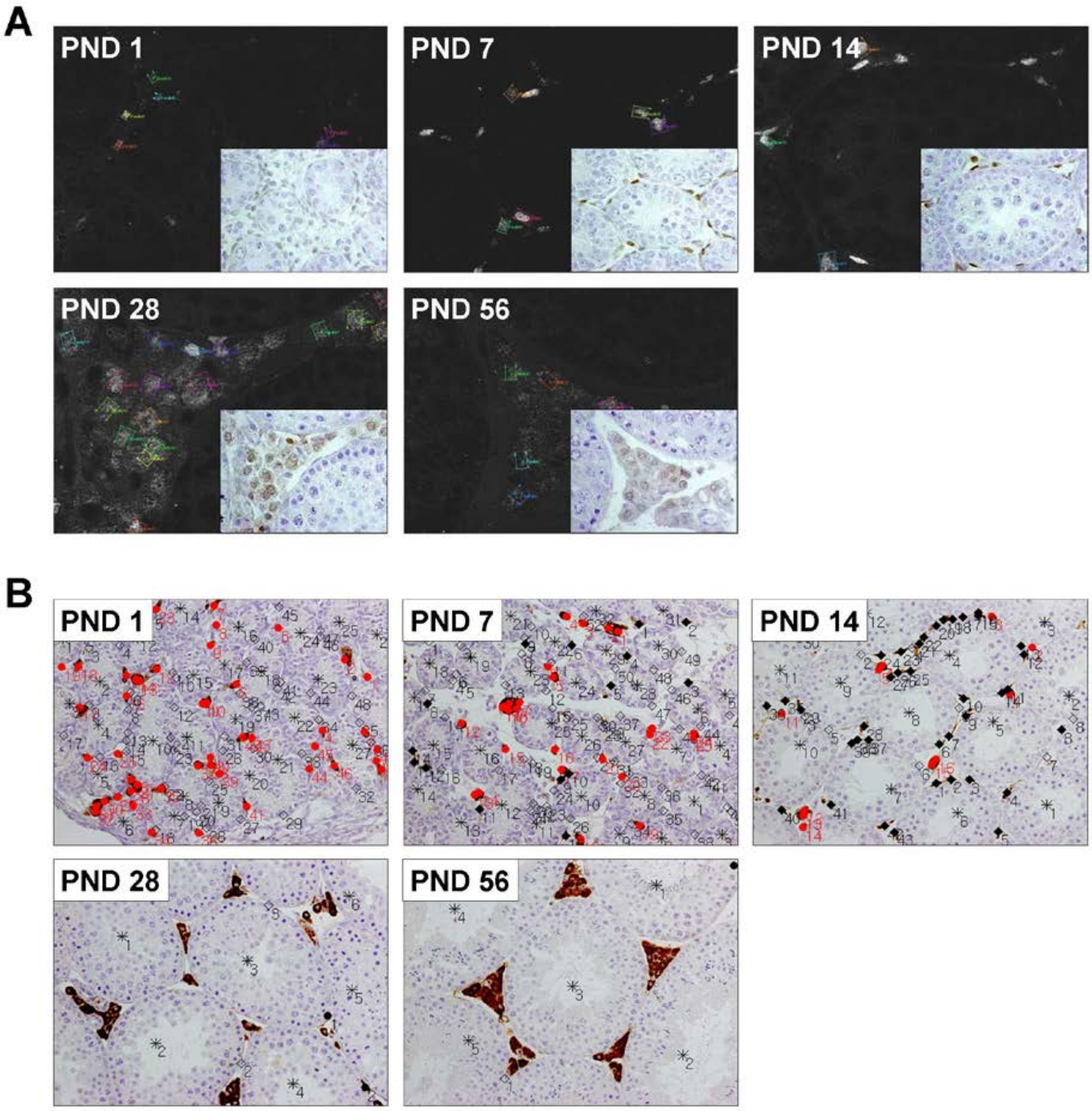

Supplementary Fig. S2. Quantitative image analysis. (A) The intensity of nuclear ESR1 immunoreactivity in Leydig cells within the P-Parallels was measured, and the mean intensity of ESR1 was calculated. (B) Counts of Leydig cells and seminiferous tubules. Red circles, HSD3B-positive round cells (FLCs); empty diamonds, HSD3B-negative spindle-shaped cells; black diamonds, HSD3B-positive spindle-shaped cells (PLCs); asterisks, seminiferous tubules.

Supplementary Table S1. Primer sequences (forward and reverse) used for RT-PCR

Name	Primer sequence
Esr1	5'-TGCCGTGTGCAATGACTATG-3'
	5'-TTTCATCATGCCCACTTCGT-3'
Esr2	5'-CAGCAGCAGTCAGTCCGTCT-3'
	5'-ACCCCGAGATTGAGGACTTG-3'
Lhcgr	5'-CGCCACGTCATCCTACTCAC-3'
	5'-TGGCAGAATAAAGCGTCTCG-3'
Star	5'-GAGGTTCCACCTGTGTGCTG-3'
	5'-CAGGTGGTTGGCGAACTCTA-3'
Cyp11a1	5'-TCAAAGCCAGCATCAAGGAG-3'
	5'-CCGGAAGTGGGTGGTATTTT-3'
Cyp17a1	5'-TAGGCTTCAGTCGAACACCG-3'
	5'-GGGATGGCAAACTCTCCAAT-3'
HSD3b6	5'-CCCAGTACCTGAGGAAAGCC-3'
	5'-TCTTCCTCGTTGCCATTCAG-3'
HSD17b3	5'-GTCCCTGGCCTCTTTACAGC-3'
	5'-TTTAACAAACTCATCGGCGG-3'
Ki67	5'-GCCAAGGGTAACTCGTGGAA-3'
	5'-GGAGGTGAAAACCACACTGG-3'
Pcna	5'-AGAAGAGGAGGCGGTAACCA-3'
	5'-ATCTTCAATCTTGGGAGCCA-3'
Cldn11	5'-GCTGGGGTGCTCCTTATTCT-3'
	5'-GGAGCAGCAGACGATGACAC-3'
Sycp3	5'-CAGAGCCAGAGAATGAAAGCA-3'
	5'-GCCATCTCTTGCTGCTGAGT-3'
Prm2	5'-ATGGTTCGCTACCGAATGAG-3'
	5'-TTAGTGATGGTGCCTCCTAC-3'
Rpl7	5'-TCAATGGAGTAAGCCCAAAG-3'
	5'-CAAGAGACCGAGCAATCAAG-3'
